# Hospital admission rates and related outcomes among adult Aboriginal australians with bronchiectasis – a ten-year retrospective cohort study

**DOI:** 10.1186/s12890-024-02909-x

**Published:** 2024-03-06

**Authors:** Timothy Howarth, Claire Gibbs, Subash S. Heraganahally, Asanga Abeyaratne

**Affiliations:** 1 Department of Technical Physics, University of Eastern Finland, Kuopio, Finland; 2 Darwin Respiratory and Sleep Health, Darwin Private Hospital, Darwin, NT Australia; 3 College of Health and Human Sciences, Charles Darwin University, Darwin, NT Australia; 4 Diagnostic Imaging Center, Kuopio University Hospital, Kuopio, Finland; 5 Department of Respiratory and Sleep Medicine, Royal Darwin Hospital, Tiwi, Darwin, NT Australia; 6 College of Medicine and Public Health, Flinders University, Darwin, NT Australia; 7 Menzies School of Health Research, Darwin, NT Australia

**Keywords:** Asthma, BMI, COPD, Chest CT, ICU, ICS, ICD, Pneumonia, Respiratory failure, Spirometry

## Abstract

**Background:**

This study assessed hospitalisation frequency and related clinical outcomes among adult Aboriginal Australians with bronchiectasis over a ten-year study period.

**Method:**

This retrospective study included patients aged ≥ 18 years diagnosed with bronchiectasis between 2011 and 2020 in the Top End, Northern Territory of Australia. Hospital admissions restricted to respiratory conditions (International Classification of Diseases (ICD) code J) and relevant clinical parameters were assessed and compared between those with and without hospital admissions.

**Results:**

Of the 459 patients diagnosed to have bronchiectasis, 398 (87%) recorded at least one respiratory related (ICD-J code) hospitalisation during the 10-year window. In comparison to patients with a recorded hospitalisation against those without—hospitalised patients were older (median 57 vs 53 years), predominantly females (54 vs 46%), had lower body mass index (23 vs 26 kg/m^2^) and had greater concurrent presence of chronic obstructive pulmonary disease (COPD) (88 vs 47%), including demonstrating lower spirometry values (forced vital capacity (FVC) and forced expiratory volume in 1 s (FEV_1_) (median FVC 49 vs 63% & FEV_1_ 36 vs 55% respectively)). The total hospitalisations accounted for 3,123 admissions (median 4 per patient (IQR 2, 10)), at a median rate of 1 /year (IQR 0.5, 2.2) with a median length of 3 days (IQR 1, 6). Bronchiectasis along with COPD with lower respiratory tract infection (ICD code-J44) was the most common primary diagnosis code, accounting for 56% of presentations and 46% of days in hospital, which was also higher for patients using inhaled corticosteroids (81 vs 52%, *p* = 0.007). A total of 114 (29%) patients were recorded to have had an ICU admission, with a higher rate, including longer hospital stay among those patients with bronchiectasis and respiratory failure related presentations (32/35, 91%). In multivariate regression model, concurrent presence of COPD or asthma alongside bronchiectasis was associated with shorter times between subsequent hospitalisations (-423 days, *p* = 0.007 & -119 days, *p* = 0.02 respectively).

**Conclusion:**

Hospitalisation rates among adult Aboriginal Australians with bronchiectasis are high. Future interventions are required to explore avenues to reduce the overall morbidity associated with bronchiectasis among Aboriginal Australians.

**Supplementary Information:**

The online version contains supplementary material available at 10.1186/s12890-024-02909-x.

## Background

Bronchiectasis is a chronic pulmonary condition characterised by a cycle of recurrent lower respiratory tract infections and airway inflammation giving rise to frequent hospitalisations and reduced quality of life [1, 2]. Globally, there is emerging evidence in the literature to suggest that presence of bronchiectasis is associated with a significant degree of hospitalisations and high mortality rates [3–6]. However, these global studies portray bronchiectasis as largely a disease of the elderly [7–9]. In the First Nations Indigenous people’s context, for the majority bronchiectasis is a lifelong disease, or at best an early adulthood to middle-age disease [10–13] (from here on “Indigenous” is used to refer to global First nations populations, while “Aboriginal Australian” is used to specifically refer to Australia’s’ First Nations people). In the paediatric Aboriginal Australian population, bronchiectasis incidence is one among the highest in the world [14, 15], and the prevalence remains significantly higher among Aboriginal Australians compared to non-Aboriginal Australian adults [16], as well as among global Indigenous populations, as observed among the New Zealand’s First Nations Indigenous Māori and Pacific Islander populations compared to New Zealand’s non-Indigenous population [17, 18].

Presence of bronchiectasis can lead to a greater number of hospitalisations and associated mortality both as direct results of bronchiectasis exacerbations per se or as an indirect contributor to other comorbidities [19–23]. Among both Aboriginal Australians and among Indigenous populations worldwide the prevalence of multimorbidity is high [24–27]. Indeed, concurrent presence of bronchiectasis and chronic obstructive pulmonary disease (COPD) has been reported in up to 50% of adult Aboriginal Australians [28–33]. Furthermore, hospitalisation frequency related to bronchiectasis, and bronchiectasis specific mortality rates appear to be significantly higher among Indigenous patients than their non-Indigenous counterparts [34–37]. However, although a higher hospitalisation frequency amongst Indigenous patients with bronchiectasis has been described in the existing literature, there is little data regarding other pertinent factors such as principal reasons for hospital presentation, length of hospital stays, utilisation of intensive care unit (ICU) or mechanical ventilation and therapeutic interventions, in particular use of inhaled pharmacotherapy. In the recent past, a significant prevalence of chronic respiratory disorders, including bronchiectasis among adult Aboriginal Australians in the Northern Territory (NT) of Australia has begun to be elucidated [10, 24]. Hence, it is timely to investigate in greater detail hospital presentations and related outcomes among adult Aboriginal Australians with bronchiectasis. Therefore, this study sets forth to describe the demographics, relevant clinical parameters, frequency and reason for hospitalisations, length of stay, treatment details, utilisation of ICU and ventilation in an adult Aboriginal Australian cohort diagnosed to have bronchiectasis over a ten-year period (2011–2020) in the Top End Health Service (TEHS) region of the NT of Australia.

## Methods

### Study setting and study population

This study was conducted at the respiratory and sleep division based at the Royal Darwin Hospital (RDH), a university affiliated tertiary care teaching hospital and Darwin Respiratory and Sleep Health, Darwin Private Hospital, within the TEHS, NT region of Australia. The TEHS region within the NT covers approximately 35% or 475,338 km^2^ of the total area of the NT and contains an estimated adult population (≥ 18 years) of 129,000 people, representing almost 80% of the total NT adult population [38, 39]. In the TEHS, 22% of the adult population are Aboriginal Australians, of whom approximately 77% reside in remote or very remote communities as defined by the Australian Statistical Geographic Standard Level 4 or Level 5 [40].

### Ethics

This study was approved by the Human Research Ethics governance/committee of the TEHS, NT and Menzies School of Health Research (Reference: HREC; 2019–3547). The authors acknowledge the rights of Australian Aboriginal people involved in this study, and as such conducted and reported according to strengthening and reporting of health research involving Aboriginal people [41], including consultations with institute Aboriginal representatives. Individual patient consent was not required, as it was a retrospective study and no active interventions were undertaken and the need for individual consent was waived by the Human Research Ethics governance/committee of the TEHS, NT and Menzies School of Health Research.

### Study participants

**Fig. 1:**
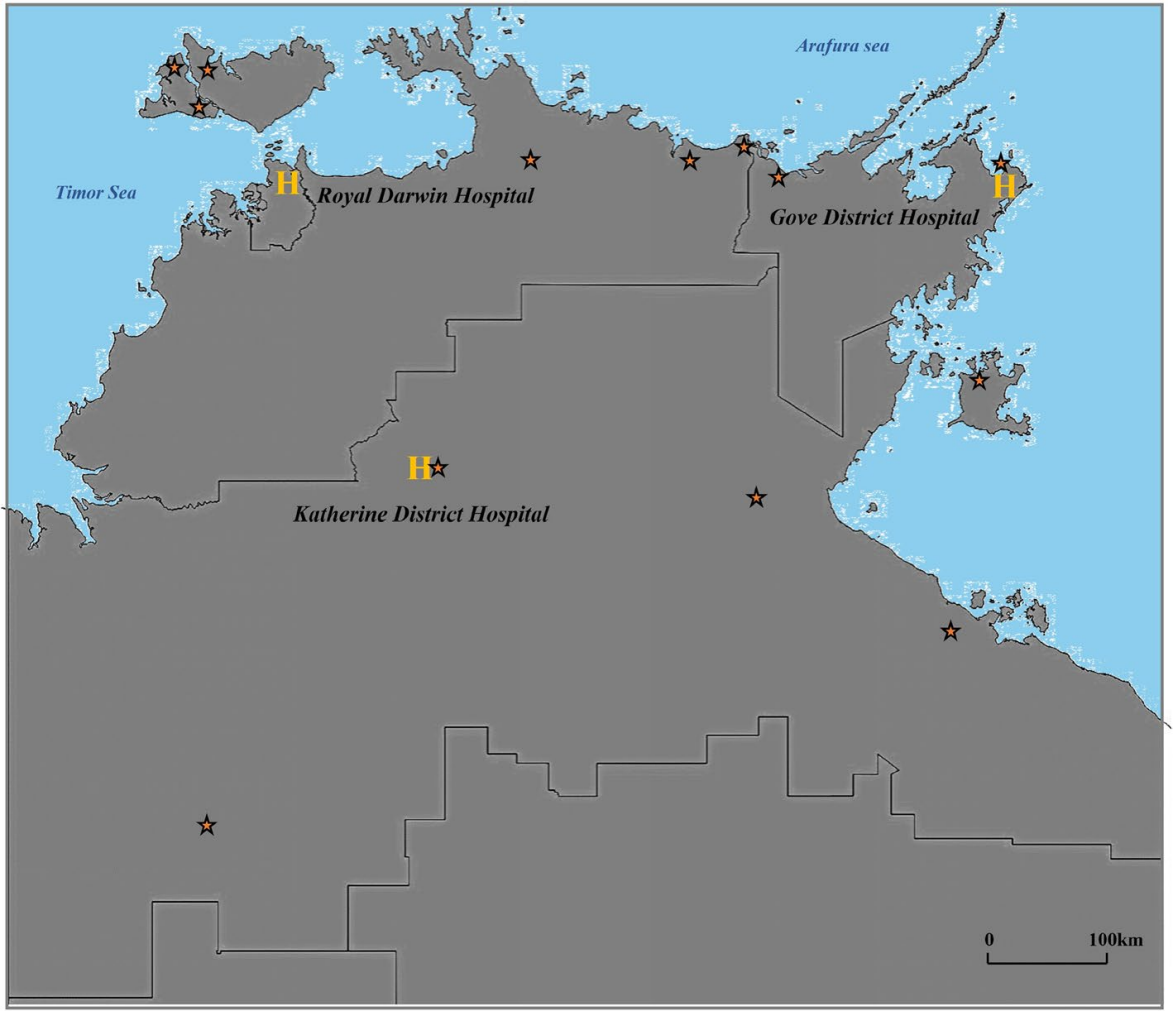
Map of Top End NT showing approximate locations of communities with ≥ 10 active bronchiectasis cases and distance from a secondary and tertiary care centre

This study is a part of a larger project examining various aspects of bronchiectasis disease profiles among the adult Aboriginal Australian population residing in the TEHS health district region of the NT of Australia, which is inclusive of all adult Australian Aboriginal patients aged ≥ 18 years identified to have bronchiectasis via chest Computed tomography (CT) scan between 2011 and 2020. Hospital admission data for this period (1 Jan 2011 – 31 Dec 2020) was assessed across the three hospitals in the TEHS regions (RDH, Katherine District Hospital and Gove District Hospital) (Fig. 1).

### Clinical data examined

Demographics, residence locality, smoking status, concurrent medical co-morbidities, including other non- bronchiectasis respiratory conditions, spirometry parameters (Forced expiratory volume in 1 s (FEV_1_), Forced vital capacity (FVC) & FEV_1_/FVC) when available and chest CT scan results for location of bronchiectasis were recorded. Hospital admissions restricted to those being respiratory related (International Classification of Diseases (ICD) code J and sub class) were included for analysis. Principal diagnosis, frequency, length of hospital stay and time between hospitalisations were assessed. Hospitalisation rate was defined as the number of hospitalisations divided by the time from the date of the first recorded hospitalisation within the study period until either the end of the study period, or death – whichever occurred first. ICU visits and mechanical ventilation data were collected at the person level and not broken down per visit. Further details on methods and study design are available form a recent report from our centre [16].

### Statistical analysis

Continuous data were displayed as median (interquartile range (IQR)) and categorical data as number (%). Differences in demographic and clinical variables between hospitalised and non-hospitalised patients were tested via quantile regression (continuous parameters) or logistic regression (categorical parameters). Differences in the proportion of patients with each specific ICD code, the proportion of total visits with that code, and the median length of stay per code between patients using and not using ICS were tested via logistic and quantile regressions respectively. Univariate and multivariate mixed regression models were used to test the effect of demographic and clinical parameters on total time spent in hospital, and median length of time between hospitalisations for patients who recorded at least two hospitalisations, reporting results as beta (95% confidence interval (CI)). Stepwise regression was utilised to define the model for the multivariate regressions. Parameters for which the entire cohort did not have complete data (BMI, lung function parameters and smoking status) were excluded from the model, while age, sex and remote/urban residence were forced inclusions, with other parameters excluded at a threshold of *p* > 0.1. A second model incorporated all parameters with forced inclusion of age, sex and remote/urban residence, and is displayed in Supplement 1. Romano-Wolff correction was utilised for multiple hypothesis adjustment, with 150 bootstrap replications. All analysis was conducted in STATA IC 15 (College Station, Texas).

## Results

### Hospital admission data

Of the 459 patients included in this database, 398 patients (87%) recorded at least one respiratory related hospitalisation during the 10-year study window until 31 December 2020, and 229 (50.1%) recorded at least one admission within the last year (median rate 1 (IQR 0, 2)) (Table 1). Two patients recorded over 100 hospitalisations during the study window, potentially skewing the results and thus were removed from further analysis. Patients who recorded a hospital admission showed similar age, gender, remoteness, BMI and smoking status distribution as those without any hospital admissions (Table 1). However, patients with a hospital admission had reduced FEV_1_ (36% predicted (IQR 27, 48.5) vs. 55% predicted (IQR 51, 63)). The prevalence of comorbid COPD was higher in the hospitalised group (88 vs. 48%, *p* = 0.029), however there was no difference in the prevalence of other comorbidities. Inhaled pharmacotherapy, including ICS prescriptions were recorded at almost twice the rate among the hospitalised patient group compared to the non-hospitalised patients (*p* = 0.029).

**Table 1:** Demographic and clinical information of patients split by whether had a hospitalisation during the study period

Clinical parameters	No admissions (*n* = 61)	Admissions (*n* = 396)	RW *p*-value
Age	52.75 (47.5, 64.56)	56.06 (48.67, 65.3)	0.882
Female	40 (65.6%)	214 (54%)	0.882
Remote / very remote	59 (96.7%)	364 (91.9%)	0.941
BMI (kg/m^2^) (*n* = 17 & 152)	26.53 (20.2, 31.6)	22.95 (18.97, 26.25)	0.588
Never smoker (*n* = 16 & 134)	2 (12.5%)	20 (14.9%)	1.000
Former smoker (*n* = 16 & 134)	8 (50%)	56 (41.8%)	1.000
Current Smoker (*n* = 16 & 134)	6 (37.5%)	58 (43.3%)	1.000
FVC (% predicted) (*n* = 17 & 152)	63 (56, 67)	49 (38.5, 63)	0.118
FEV_1_ (% predicted) (*n* = 17 & 152)	55 (51, 63)	35.95 (27, 48.5)	0.029
FEV_1_/FVC (*n* = 17 & 152)	0.77 (0.65, 0.82)	0.65 (0.48, 0.76)	0.235
**Respiratory co-morbidities**			
COPD	29 (47.5%)	349 (88.1%)	0.029
Asthma	11 (18%)	106 (26.8%)	0.912
Lung cancer	3 (4.9%)	15 (3.8%)	1.000
ILD	0 (0%)	3 (0.8%)	-
**Other medical co-morbidities**			
Hypertension	37 (60.7%)	250 (63.1%)	1.000
Type 2 diabetes	28 (45.9%)	194 (49%)	1.000
Chronic kidney disease	27 (44.3%)	157 (39.6%)	1.000
CAD	17 (27.9%)	142 (35.9%)	0.971
HF	4 (6.6%)	35 (8.8%)	1.000
**Bronchiectasis chest CT location**			
LLL	43 (70.5%)	293 (74%)	1.000
RLL	35 (57.4%)	249 (62.9%)	1.000
RML	30 (49.2%)	164 (41.4%)	0.971
RUL	18 (29.5%)	108 (27.3%)	1.000
Lingula	18 (29.5%)	110 (27.8%)	1.000
LUL	11 (18%)	89 (22.5%)	1.000
Bilateral	39 (63.9%)	257 (64.9%)	1.000
**Medications**			
SABA	28 (45.9%)	256 (64.6%)	0.118
SAMA	0 (0%)	32 (8.1%)	-
LABA	21 (34.4%)	256 (64.6%)	0.029
LAMA	14 (23%)	197 (49.7%)	0.059
ICS	18 (29.5%)	232 (58.6%)	0.029

*p*-values derived from quantile regression (continuous parameters) or logistic regression (categorical parameters), with additional adjustment for multiple comparisons via Romano-Wolf correction

BMI, smoking status and lung function parameters were only available in a subset of patients, with the number available lifted in the leftmost column

*Abbreviations***:** *BMI* Body mass index, *FVC* Forced vital capacity, *FEV*_*1*_ Forced expiratory volume in one second, *COPD* Chronic obstructive pulmonary disease, *ILD* Interstitial lung disease, *HF* Heart failure, *CAD* Coronary artery disease, *LLL* Left lower lobe, *RLL* Right lower lobe, *RML* Right middle lobe, *RUL* Right upper lobe, *LUL* Left upper lobe, *RW-p* Romano-Wolff *p*-value, *SABA *Short acting beta agonist, *LABA* long acting beta agonist, *SAMA *short acting muscarinic agent, *LAMA* long acting muscarinic agent, *ICS *inhaled corticosteroids.

### Length of hospital admission data

A total of 3,123 hospital admissions were available to be assessed, with patients recording a median 4 (IQR 2, 10) admissions over the study period. The length of time from patients first admission until the end of the study period or death was a median 5.4 years (IQR 3.2, 8.1), resulting in an overall median hospital admission rate of 1 /year (IQR 0.5, 2.2). The median length of individual hospital admissions was 3 days (IQR 1, 6), with 390 (12.5%) same day separations and 162 (5.2%) of greater than two weeks duration. In total, patients with at least one hospital admission spent a median 21 days (IQR 9, 52) in hospital across the study period. At least two hospitalisations were recorded for 327/396 (82%) patients. Of the 2,727 hospital admissions among these patients 926 (34%) occurred within 30 days of a prior hospitalisation, 349 (12.8%) within 30–60 days from a prior hospitalisation and the remaining 1,452 (53.3%) more than 60 days after.

### Respiratory related hospital admission as per ICD separation codes

Hospital admissions coded J44 (COPD with lower respiratory tract infection) were the most common, with 69.2% of patients recording at least one J44 visit (Table 2) (the description of individual ICD codes is detailed in supplementary file 2). Furthermore, J44 visits accounted for 55.7% of hospital presentations and 46% of total days in hospital. Pneumonia related visits (J15) and those related to respiratory failure (J96) however were associated with the longest stays in hospital per visit (J15, median 6 days (IQR 3, 10.5) and J96, median 7 days (IQR 4, 12)).

**Table 2:** Number of hospitalisations and duration as per International Classification of Diseases codes

ICD separation codes	Patients with this code (*n* = 396)	Number of visits with this code (*n* = 3045)	Total days in hospital (*n* = 14,008)	Median days in hospital per episode	Single day separations
J6	9 (2.3%)	12 (0.4%)	22 (0.2%)	1 (0.5, 2.5)	3 (25%)
J10	71 (17.9%)	82 (2.7%)	440 (3.1%)	4 (3, 6)	4 (4.9%)
J11	12 (3%)	12 (0.4%)	58 (0.4%)	4 (1.5, 7)	0 (0%)
J13	40 (10.1%)	51 (1.7%)	325 (2.3%)	5 (3, 8)	0 (0%)
J14	42 (10.6%)	49 (1.6%)	219 (1.6%)	4 (2, 6)	6 (12.2%)
J15	68 (17.2%)	108 (3.5%)	1008 (7.2%)	6 (3, 10.5)	5 (4.6%)
J18	173 (43.7%)	383 (12.6%)	1904 (13.6%)	4 (2, 6)	18 (4.7%)
J22	72 (18.2%)	122 (4%)	363 (2.6%)	2 (1, 4)	27 (22.1%)
J44	274 (69.2%)	1697 (55.7%)	6439 (46%)	3 (1, 5)	257 (15.1%)
J45	20 (5.1%)	64 (2.1%)	113 (0.8%)	1 (1, 2)	13 (20.3%)
J47	136 (34.3%)	364 (12%)	1980 (14.1%)	3 (2, 6)	36 (9.9%)
J69	12 (3%)	25 (0.8%)	349 (2.5%)	6 (4, 10)	1 (4%)
J85	10 (2.5%)	18 (0.6%)	240 (1.7%)	6.5 (4, 13)	2 (11.1%)
J96	35 (8.8%)	46 (1.5%)	472 (3.4%)	7 (4, 12)	1 (2.2%)
J98	12 (3%)	12 (0.4%)	76 (0.5%)	2 (0.5, 7)	3 (25%)

ICD codes with fewer than 10 associated hospital presentations were omitted from this table

Single day separations utilise the total number of visits with that code as the denominator for each cell

### Hospital admission and Inhaled pharmacotherapy use data

Among the patients with at least one hospital admission, 232 (58.6%) had recorded use of ICS. Among these 188/232 (81%) recorded at least one J44 related hospitalisation, a greater proportion than among those patients not using ICS 86/164 (52.4%) (RW *p*-value = 0.007). There were no differences in the proportion of patients presenting with other codes, the total number of presentations for any codes, nor the length of time spent in hospital between ICS and non-ICS use.

### ICU admission data

Of the 396 patients with a hospital admission, 114 (28.8%) had at least one ICU admission, among whom the median total number of hours spent in ICU was 128 h (IQR 68, 236) (Supplementary file 3). A greater proportion of patients with a J96 code recorded an ICU admission (91.4%) compared to any other code. Patients who recorded at least one J85 (median 440 h (IQR 7, 652)) or J96 (median 215 h (IQR 106, 278.5)) hospital admission recorded more hours in ICU than other patients. Mechanical ventilation was recorded for 32 patients (8.1%), for whom the median total number of hours on ventilation was 48.5 h (IQR 30.5, 145.5). Almost half (43%) of patients with a J96 code recorded mechanical ventilation.

### Regression analysis

In univariate regression models each 10% decrease in FEV_1_ was associated with a 6.33-day (95% CI 3.43, 9.23) increase in total time spent in hospital, while each 10% decrease in FVC was associated with a 6-day (95% CI 2.89, 9.12) increase. Comorbid COPD (19-days (95% CI 10.13, 27.87)), asthma (13-days (95% CI 6.18, 19.82)), and heart failure (23-days (95% CI 12.33, 33.67)) were each associated with increases in the total number of days spent in hospital (Table 3).

**Table 3:** Univariate and multivariate regression for the total number of days spent in hospital among those patients with at least one hospital admission

Clinical parameters	Univariate model	RW p	Multivariate model	RW p
Age	-0.25 (-0.58, 0.07)	0.974	-0.44 (-0.71, -0.17)	0.497
Female	-3 (-9.48, 3.48)	1.000	-2.72 (-9.03, 3.58)	0.993
Urban	-1 (-13.45, 11.45)	1.000	0.91 (-10.75, 12.58)	1.000
BMI (*n* = 152)	-0.64 (-1.58, 0.31)	1.000		
Former smoker	-8 (-26.05, 10.05)	1.000		
Current Smoker	-3 (-21.05, 15.05)			
FVC (10% predicted) (*n* = 152)	6 (2.88, 9.12)	0.828		
FEV_1_ (10% predicted) (*n* = 152)	6.33 (3.43, 9.23)	0.729		
FEV_1_/FVC (*n* = 152)	0.11 (-0.26, 0.47)	1.000		
COPD	19 (10.13, 27.87)	0.689	12.88 (2.28, 23.48)	0.676
Asthma	13 (6.18, 19.82)	0.517		
ILD	6 (-34.35, 46.35)	1.000		
Lung cancer	0 (-16.03, 16.03)	1.000		
Hypertension	4 (-3.27, 11.27)	0.934	10.13 (3.32, 16.93)	0.563
Type 2 diabetes	5 (-1.71, 11.71)	0.993		
Chronic kidney disease	0 (-6.39, 6.39)	0.993		
CAD	6 (-0.15, 12.15)	0.974		
HF	23 (12.33, 33.67)	0.636	15.36 (4.4, 26.32)	0.583
RLL	5 (-1.75, 11.75)	0.993		
RML	-7 (-13.43, -0.57)	0.974	-9.06 (-15.71, -2.41)	0.636
RUL	6 (-1.17, 13.17)	0.755	11.98 (4.77, 19.2)	0.483
LLL	2 (-5, 9)	1.000		
Lingula	1 (-5.95, 7.95)	1.000		
LUL	6 (-1.57, 13.57)	0.894		
Bilateral	5 (-1.58, 11.58)	0.993		
SABA	9 (2.25, 15.75)	0.848		
SAMA	10 (-2.49, 22.49)	0.993		
LABA	15 (8.41, 21.59)	0.623	9.44 (2.38, 16.49)	0.636
LAMA	15 (8.81, 21.19)	0.397		
ICS	12 (5.97, 18.03)	0.682		

Multivariate model created via stepwise regression with forced inclusion of demographic variables (age, sex and residence location) and excluding smoking status, BMI and lung function parameters due to incomplete data, with exclusion from the model set at *p* > 0.01

*Abbreviations*: *BMI* Body mass index, *FVC* Forced vital capacity, *FEV*_*1*_ Forced expiratory volume in one second, *COPD* Chronic obstructive pulmonary disease, *ILD* Interstitial lung disease, *HF* Heart failure, *CAD* Coronary artery disease, *LLL* Left lower lobe, *RLL* Right lower lobe, *RML* Right middle lobe, *RUL* Right upper lobe, *LUL* Left upper lobe, *SABA* Short acting beta agonists, *SAMA* Short acting muscarinic agents, *LABA* Long acting beta agonists, *LAMA* Long acting muscarinic agents, *ICS* Inhaled corticosteroids, *RW-p* Romano-Wolff *p*-value

In the multivariate model, comorbid COPD (12.88-days (95% CI 2.28, 23.48)), HTN (10.13-days (95% CI 3.32, 16.94)) and heart failure (15.36-days (95% CI 4.40, 26.32)) were the factors associated with increased time in hospital (Table 4). Statistical significance for all associations however was attenuated following Romano-Wolff correction.

**Table 4:** Univariate and multivariate mixed regression effects for the length of time between hospitalisations

Clinical parameters	Univariate model	RW p	Multivariate model	RW p
Age	2.38 (-1.5, 6.26)	0.656	3.65 (-0.6, 7.91)	0.205
Female	50.63 (-41.15, 142.41)	0.755	54.17 (-40.4, 148.74)	0.715
Urban	118.19 (-50.19, 286.56)	0.477	65.28 (-105.01, 235.58)	0.980
BMI (*n* = 17 & 152)	2.96 (-7.53, 13.45)	1.000		
Former smoker	17.02 (-210.74, 244.79)	1.000		
Current Smoker	-40.72 (-267.94, 186.49)			
FVC (10% predicted) (*n* = 152)	-6.74 (-53.8, 40.31)	1.000		
FEV_1_ (10% predicted) (*n* = 152)	-30.5 (-75.27, 14.26)	0.517		
FEV_1_/FVC (*n* = 152)	1.2 (-2.64, 5.05)	0.993		
COPD	-475.9 (-649.31, -302.5)	0.007	-420.37 (-598.65, -242.09)	0.007
Asthma	-129.22 (-229.09, -29.35)	0.007	-118.83 (-222.06, -15.6)	0.020
ILD	-254.09 (-1052.65, 544.47)	1.000		
Lung cancer	-37.24 (-279.71, 205.23)	1.000	-23.87 (-259.45, 211.72)	1.000
Hypertension	-63.74 (-159.52, 32.05)	0.536	-49.31 (-154.01, 55.4)	0.894
Type 2 diabetes	-62.39 (-153.92, 29.13)	0.517		
Chronic kidney disease	4.88 (-88.86, 98.62)	1.000	-2.28 (-102.58, 98.01)	1.000
CAD	-46.85 (-141.24, 47.54)	0.861	-35.68 (-135.2, 63.84)	0.980
HF	-150.97 (-312.96, 11.02)	0.133	-111.82 (-270.71, 47.06)	0.470
RLL	-154.79 (-249.15, -60.42)	0.007		
RML	19.41 (-73.96, 112.79)	1.000	32.2 (-68.15, 132.55)	0.993
RUL	-86.13 (-187.44, 15.17)	0.212	-121.7 (-243.85, 0.44)	0.099
LLL	-41.57 (-146.7, 63.55)	0.980	-19.69 (-142.34, 102.96)	1.000
Lingula	-0.09 (-102.46, 102.27)	1.000		
LUL	9.14 (-99.88, 118.16)	1.000	95.49 (-36.73, 227.71)	0.437
Bilateral	-83.82 (-181.04, 13.39)	0.199	-55.44 (-171.64, 60.76)	0.894
SABA	-58.67 (-156.22, 38.88)	0.676		
SAMA	-30.2 (-193.6, 133.21)	1.000		
LABA	-94.12 (-192.92, 4.69)	0.119		
LAMA	-79.83 (-171.3, 11.64)	0.192		
ICS	-56 (-150.35, 38.35)	0.695		

Multivariate model created via stepwise regression with forced inclusion of demographic variables (age, sex and residence location) and excluding smoking status, BMI and lung function parameters due to incomplete data, with exclusion from the model set at *p* > 0.01

*Abbreviations*: *BMI* Body mass index, *FVC* Forced vital capacity, *FEV*_*1*_ Forced expiratory volume in one second, *COPD* Chronic obstructive pulmonary disease, *ILD* Interstitial lung disease, *HF* Heart failure, *CAD* Coronary artery disease, *LLL* Left lower lobe, *RLL* Right lower lobe, *RML* Right middle lobe, *RUL* Right upper lobe, *LUL* Left upper lobe, *RW-p* Romano-Wolff *p*-value, *SABA* Short acting beta agonists, *SAMA* Short acting muscarinic agents, *LABA* Long acting beta agonists, *LAMA* Long acting muscarinic agents, *ICS* Inhaled corticosteroids

### Regression analysis for patients with two or more hospital admissions

At least two hospitalisations were recorded for 327/396 (82%) patients. In univariate mixed regression models, there was reduced length of time between hospitalisations among patients with comorbid COPD (-475.9 days (95% CI -649.31, -302.5)), asthma (-129.22 days (95% CI -229.09, -29.35)), and with right lower lobe involvement (-154.79 days (95% CI -249.15, -60.42)), (Table 4). In the multivariate model, comorbid COPD (-423.37 days (95% CI -598.65, -2242.09)) and asthma (-118.83 days (95% CI -222.06, -15.6)) remained associated with reduced interval between hospitalisations.

## Discussion

In this study, 87% of bronchiectasis patients were noted to have had at least one respiratory related hospital admission during the 10-year study period, with 29% requiring ICU admission. Patients spent a median of 3 weeks in hospital, and 82% of patients had recurrent admissions. Patients who required hospitalisation appeared to be in poorer health than other patients as evidenced by a higher rate of comorbidities, lower lung function values and greater prescription rates of inhaled pharmacotherapy. Presence of COPD or asthma were associated with greater lengths of time spent in hospital, and shorter intervals between hospitalisations.

In the global non-Indigenous populations, hospital admission data and factors associated with mortality, including the economic burden secondary to bronchiectasis are well documented [3–7, 19–23]. Among the adult Indigenous population however, literature surrounding hospital admissions is sparse – despite a higher burden of chronic respiratory disease [10, 24]. On the backdrop of poorer socioeconomic environment, alongside geographic, social and systemic barriers to healthcare, Indigenous people typically display a greater degree of multimorbidity, which in turn may alter the way in which respiratory diseases manifest in comparison to their non-Indigenous counterparts [28, 31, 42–48].

The majority of patients (69.4%) had an admission secondary to COPD (J44), similar to what has been observed in previous reports [49]. Although, indeed it is difficult to ascertain if bronchiectasis is a primary or a secondary factor driving hospital admission rates in the presence of multi-respiratory morbidity, including concurrent presence of bronchiectasis and COPD among Aboriginal Australians [28], however, it is reasonable to speculate that the presence of bronchiectasis among patients with COPD would have perpetuated exacerbation rates and subsequent hospitalisations. Previous studies in other population settings have demonstrated worse outcomes when bronchiectasis co-exists with COPD, especially in the presence of a smoking history, alongside lower lung function parameters [3, 5]. Indeed, in the current cohort with hospitalisations, COPD was recorded in 88%, a smoking history in 85%, and median FVC & FEV_1_ of 49 & 36% predicted respectively. Furthermore, a 10% reduction in FEV_1_ was associated with a mean 5.3 day increase in the total number of days in hospital, although significance was attenuated on multiple hypotheses correction. Previous reports have shown that among adult Aboriginal Australians, lung function parameters are substantially lower compared to their Caucasian counterparts [50–54], thus, it may be indicative that presence of lower lung function parameters could be a predictor for increased length of hospital admissions among Indigenous patients more generally, including patients with bronchiectasis. Therefore, it is reasonable to speculate that improving lung function parameters with targeted interventions among Indigenous patients with respiratory disorders will reduce hospital admission rates and length of hospitalisation. Furthermore, Bronchiectasis and COPD overlap syndrome (BCOS) is increasingly recognized [55]. Although both these conditions share several similar clinical features, the management of these conditions differs; hence, in clinical practice, differentiating if COPD or bronchiectasis is the primary disorder, especially when inhaled pharmacotherapy is considered, is vital. In addition to classical clinical manifestations, chest CT scan finding will be of heightened value in differentiating which of these conditions is predominant [56].

Among patients with bronchiectasis, a lower BMI is reported to be an independent predictor of mortality [57]. In our study we observed BMI to be lower among patients with hospital admissions– though we did not test the effect of BMI on mortality specifically in this study. If a lower BMI among Aboriginal Australian patients would translate to poorer long-term outcomes is not known currently and only prospective studies will elucidate this aspect. However, it is noteworthy that the median BMI in the current cohort is lower than the BMI reported previously among non-Aboriginal Australian cohort with bronchiectasis [58].

It is vital to note that the majority of patients in this study resided in remote/rural localities (92%) (Fig. 1). Hence, during exacerbations of airway disease and when needing hospital admissions, patients are inevitably retrieved to secondary or tertiary care centres, therefore incurring substantial cost to the health system [59, 60]. Nevertheless, the economic burden of managing patients with bronchiectasis in this region is not exactly known, especially for adult Aboriginal Australian people residing in remote and rural communities [61]. However, a study from Brazil estimated R$28 million (approx. AUD 14 million) in a twelve-year period for an average hospital stay of 8.1 days [21]. It is reasonable to speculate the economic cost could be substantially higher in the Australian setting, especially when considering transporting patients from remote communities by air and relocating family and caregivers. Due to limited access to specialist health care in remote Aboriginal Australian communities the threshold to transfer to a tertiary institution during exacerbation of airway disease may be low. This may be the reason for observing only 12.5% of hospitalisations were recorded as a same day separation. Hence, it is apparent that addressing bronchiectasis and other respiratory diseases at the community and primary health care level, may be an avenue to prevent recurrent hospital admissions and associated health care utilisation and expenditure [62]. Moreover, patients are more likely to show adherence to treatment interventions for disease management with continuity of care at community and primary health care level.

Although currently literature portrays a high bronchiectasis disease burden and associated adverse health outcomes globally, innovative preventative or therapeutic interventions are negligible, other than the proven benefits of chest physiotherapy/airway clearance techniques and classic mucolytic/mucoactive agents [63, 64]. In this study we did not explore specific treatment interventions, either in the ambulatory setting or during acute hospital admissions that would have any influence on recurrent hospital admission rates or for the length of stay. However, among patients prescribed ICS, we observed a higher proportion had an admission related to ICD code J44 compared to patients with no ICS prescription. Utilisation of ICS among patients with bronchiectasis is controversial and may be detrimental [65]. Previous studies from our centre had reported higher use of ICS among Indigenous patients with bronchiectasis and to have a significant decline in lung function parameters with ICS [66, 67]. It is unclear if ICS is over prescribed due to concurrent presence of other airway diseases such as COPD and asthma in this cohort. However, prospective studies are needed to define if increased hospital rates are related to natural disease progression and recurrent exacerbation or if they are related to aftermath effects of ICS use [68].

This study has demonstrated that the bronchiectasis disease burden is substantial, as is its impact on hospital admission rates and overall poor outcomes among adult Aboriginal Australians. In the largely non-Aboriginal Australian bronchiectasis registry, 29% of patients recorded a respiratory hospitalisation in the last year [58], while in the current Aboriginal Australian cohort, 50% did so. Moreover, despite evidence in the literature dating back to the 1980’s to suggest hospital admission rates are substantially higher among Aboriginal Australian patients in comparison to their non-Aboriginal counterparts with bronchiectasis [69], hospital admission rates continue to remain substantially high in this population, as evident form our current study. Furthermore, there has been no substantial interventions to address this disparity in the last three decades [70]. Therefore, it is apparent that it is inevitable that the noted higher morbidity secondary to bronchiectasis is likely to continue to show a grim trend for the future, unless clinically and culturally appropriate interventions are implemented urgently to address the overall respiratory disease burden among adult Aboriginal Australians, so that, in particular, bronchiectasis does not continue to remain a neglected chronic lung disease among Aboriginal Australians for the foreseeable future [71].

### Limitations

This study’s outcomes pertain to Aboriginal Australian people residing in the TEHS region of the NT of Australia and the results represented in this study cannot be generalised to the wider Aboriginal populations in Australia or for Indigenous people globally. We did not assess patients’ presentations to primary health care or at remote community health centres with exacerbation of their airway disease and interventions undertaken, as this was beyond the scope of this study. We also did not have data to represent therapeutic interventions during hospital admissions, which would have provided more insight for those patients with recurrent hospital admissions, especially if interventions such as chest physiotherapy/sputum clearance techniques were implemented. Moreover, with a higher presence of COPD in this population, it is uncertain if bronchiectasis is a primary or a secondary cause for hospital admissions. Furthermore, there may be a bias for those patients recorded to have no hospital admissions, as it is possible despite exacerbations of airway disease, due to remoteness and geographical isolations they did not present to hospital for admission and also spirometry data was not available in all patients. In addition, as this study was retrospective in nature, this would have introduced potential bias in the outcomes observed. Nonetheless, this is the first study to represent hospital admission data in an adult Aboriginal Australian cohort, in this region. The outcomes represented in this study may be an avenue for health organisations and stakeholders to implement strategies to address to close the respiratory health gap among adult Aboriginal Australians and Indigenous people globally.

## Conclusion

This study has demonstrated that respiratory related hospital and ICU admission rates and frequency are alarmingly high among adult Aboriginal Australians secondary to bronchiectasis. Concurrent presence of asthma, COPD, heart failure and reduced lung function parameters are associated with more time spent during hospital admission. The majority of recurrent hospitalisations were observed in the presence of underlying COPD or asthma. Future interventions are required at primary/community health care level to prevent recurrent hospitalisations, ongoing further morbidity and mortality among adult Aboriginal Australians suffering from bronchiectasis.

### Supplementary Information

**Supplementary Material 1.**

**Supplementary Material 2.**

**Supplementary Material 3.**

## Data Availability

All data generated or analyzed during this study are included in this published article.

## Abbreviations

BMI: Body mass index
COPD: Chronic obstructive pulmonary disease
CI: Confidence interval
CT: Computed tomography
FVC: Forced vital capacity
FEV_1_: Forced expiratory volume in 1 s
HTN: Hypertension
ICD: International Classification of Diseases
ICU: Intensive care unit
IQR: Interquartile range
NT: Northern Territory
RDH: Royal Darwin hospital
TEHS: Top End Health Service
