# Supplementary material for: Hospital admission rates and related outcomes among adult Aboriginal australians with bronchiectasis – a ten-year retrospective cohort study
Source: BMC Pulm Med. 2024 Mar 6;24:118. doi: 10.1186/s12890-024-02909-x (PMC10918854; doi:10.1186/s12890-024-02909-x)
Supplement: Supplementary file 1 — Supplementary Material 1. [file 12890_2024_2909_MOESM1_ESM.docx]

**Supplement 1:** Multivariate stepwise regression models including BMI, FVC and FEV_1_ for the total days spent in hospital for patients with at least one admission, and length of time (in days) between hospitalisations for patients with at least two admissions.

|  | **Days spent in hospital** |  | **Length of time between hospitalisations** |  |
| --- | --- | --- | --- | --- |
| **Clinical parameters** | **Model 1** | **RW p** | **Model 1** | **RW p** |
| Age | -0.42 (-0.87, 0.02) | 0.868 | 1.53 (-5.46, 8.52) | 1.000 |
| Female | -4.94 (-15.17, 5.28) | 0.993 | -41.34 (-200.39, 117.7) | 1.000 |
| Urban | 0.23 (-19.97, 20.44) | 1.000 | -76.73 (-388.16, 234.69) | 1.000 |
| FVC (10% predicted) (n=152) |  |  | 42.21 (-26.37, 110.79) | 0.656 |
| FEV_1_ (10% predicted) (n=152) | 5.34 (2.34, 8.34) | 0.470 | -62.68 (-130.79, 5.43) | 0.146 |
| Asthma | 11.51 (-0.47, 23.49) | 0.868 |  |  |
| Lung cancer |  |  | -132.19 (-438.87, 174.49) | 0.934 |
| Hypertension | 17.8 (6.92, 28.67) | 0.530 |  |  |
| Chronic kidney disease |  |  | 15.33 (-141.74, 172.39) | 1.000 |
| CAD |  |  | -64.63 (-226.23, 96.97) | 0.980 |
| HF |  |  | -232.22 (-555.9, 91.45) | 0.444 |
| RML |  |  | 117.84 (-57.58, 293.25) | 0.530 |
| Lingula |  |  | 25.91 (-163.97, 215.79) | 1.000 |
| SABA |  |  | 92.62 (-76.77, 262.01) | 0.768 |
| ICS |  |  | 43.45 (-139.53, 226.42) | 1.000 |
| Model 1 created via stepwise regression with forced inclusion of demographic variables (age, sex and residence location) and including all other variables, with exclusion from the model set at p>0.01  **Abbreviations:** FVC, Forced vital capacity; FEV_1_, Forced expiratory volume in one second; HF, Heart failure; CAD, Coronary artery disease; RML, Right middle lobe; RW-p, Romano-Wolff p-value | | | | |
