# Supplementary material for: Hospital admission rates and related outcomes among adult Aboriginal australians with bronchiectasis – a ten-year retrospective cohort study
Source: BMC Pulm Med. 2024 Mar 6;24:118. doi: 10.1186/s12890-024-02909-x (PMC10918854; doi:10.1186/s12890-024-02909-x)
Supplement: Supplementary file 2 — Supplementary Material 2. [file 12890_2024_2909_MOESM2_ESM.docx]

| **ICD separation codes** | **Respiratory condition** |
| --- | --- |
| J6 | Acute upper respiratory infections |
| J10 | Influenza due to other identified influenza virus with other respiratory manifestations |
| J11 | Influenza due to other identified influenza virus with other respiratory manifestations |
| J13 | Pneumonia due to Streptococcus pneumoniae |
| J14 | Pneumonia due to Hemophilus influenzae |
| J15 | Bacterial pneumonia, not elsewhere classified |
| J18 | Pneumonia, unspecified organism |
| J22 | Other acute lower respiratory infections |
| J44 | Chronic obstructive pulmonary disease with acute lower respiratory infection |
| J45 | Asthma |
| J47 | Bronchiectasis |
| J69 | Aspiration pneumonia |
| J85 | Abscess of lung and mediastinum |
| J96 | Respiratory failure |
| J98 | Other respiratory disorders |

**Supplemental file 1.** Description for ICD codes.
