# Supplementary material for: Hospital admission rates and related outcomes among adult Aboriginal australians with bronchiectasis – a ten-year retrospective cohort study
Source: BMC Pulm Med. 2024 Mar 6;24:118. doi: 10.1186/s12890-024-02909-x (PMC10918854; doi:10.1186/s12890-024-02909-x)
Supplement: Supplementary file 3 — Supplementary Material 3. [file 12890_2024_2909_MOESM3_ESM.docx]

**Supplementary file 3.** Number of patients with Intensive care unit admission and mechanical ventilation.

| **ICD separation codes** | **Number of patients with this code (n=396)** | **Number of patients with this code, and with ICU admission** | **Total hours in ICU** | **Number of patients with this code, and with mechanical ventilation** | **Total hours on ventilation** |
| --- | --- | --- | --- | --- | --- |
| J6 | 9 (2.3%) | 3 (33.3%) | 64, 127, 325 | 0 (0%) | - |
| J10 | 73 (18.4%) | 26 (35.6%) | 137.5 (69.5, 255) | 5 (6.8%) | 49 (31, 74) |
| J11 | 12 (3%) | 5 (41.7%) | 45 (35, 127) | 0 (0%) | - |
| J13 | 40 (10.1%) | 14 (35%) | 133.5 (62, 264) | 4 (10%) | 17, 70, 121, 235 |
| J14 | 43 (10.9%) | 17 (39.5%) | 213 (64, 262) | 3 (7%) | 121, 131, 291 |
| J15 | 75 (18.9%) | 36 (48%) | 169.5 (76, 266) | 8 (10.7%) | 61 (23, 176.5) |
| J18 | 174 (43.9%) | 57 (32.8%) | 98 (62, 230) | 20 (11.5%) | 41 (27.5, 181.5) |
| J22 | 73 (18.4%) | 16 (21.9%) | 145 (76, 209) | 2 (2.7%) | 235, 1045 |
| J44 | 275 (69.4%) | 84 (30.5%) | 128 (70.5, 229) | 22 (8%) | 39.5 (31, 129) |
| J45 | 20 (5.1%) | 5 (25%) | 230 (81, 287) | 2 (10%) | 129, 291 |
| J47 | 136 (34.3%) | 37 (27.2%) | 188 (87, 244) | 8 (5.9%) | 95.5 (47, 182) |
| J69 | 12 (3%) | 4 (33.3%) | 59, 195, 236, 514 | 1 (8.3%) | 31 |
| J85 | 10 (2.5%) | 6 (60%) | 440 (7, 652) | 1 (10%) | 1045 |
| J96 | 35 (8.8%) | 32 (91.4%) | 215 (106, 278.5) | 15 (42.9%) | 70 (31, 139) |
| J98 | 12 (3%) | 7 (58.3%) | 98 (77, 207) | 0 (0%) | - |
| ICD codes with fewer than 10 associated hospital presentations were omitted from this table.  Data displayed as number (%) or median (IQR). For cells with <5 participants total hours in ICU / on ventilation were written out in full. | | | | | |
